# Basic Psychological Need Satisfaction in Leisure Activities and Adolescents’ Life Satisfaction

**DOI:** 10.1007/s10964-012-9776-5

**Published:** 2012-05-25

**Authors:** Ingrid Leversen, Anne G. Danielsen, Marianne S. Birkeland, Oddrun Samdal

**Affiliations:** 1 Department of Health Promotion and Development, Faculty of Psychology, University of Bergen, P.O. Box 7807, 5020 Bergen, Norway; 2 Department of Education, Faculty of Humanities, Social Sciences, and Education, University of Tromsø, 9037 Tromsø, Norway

**Keywords:** Adolescents, Participation in leisure activities, Need satisfaction, Life satisfaction

## Abstract

Participation in leisure activities is an important arena for the positive psychological development of adolescents. The present study set out to examine the relationship between adolescents’ satisfaction of the psychological needs for competence, relatedness, and autonomy in their participation in leisure activities and their perceived life satisfaction. The aim was to identify the extent to which satisfaction of the three needs explained the relationship between participation in leisure activities and life satisfaction. These proposed mechanisms were based on previous empirical work and the theoretical frameworks of self-determination theory, and were tested in a nationally representative sample of Norwegian adolescents (*N* = 3,273) aged 15 and 16 years (51.8 % boys). The structural equation analysis showed that competence and relatedness satisfaction fully mediated the association between participation in activities and life satisfaction. Autonomy satisfaction had a direct positive effect on life satisfaction but did not show any mediation effect. The positive processes of psychological need satisfaction, and especially the need for competence and relatedness, experienced in the leisure activity domain thus seem to be beneficial for adolescents’ well-being. These findings add to previous research investigating the positive impact of need satisfaction in other important domains in the lives of children and adolescents.

## Introduction

Individuals are likely to thrive and be motivated in settings that fit well with their psychological needs (Eccles and Roeser 2011; Deci and Ryan 2000). During adolescence, individuals have an increasingly higher need for complex tasks and to take part in decision making (Eccles et al. 1993). In addition, adolescents have a higher need to be related to and supported by peers and adults other than their parents (Eccles and Roeser 2009; Oberle et al. 2011). The investigation of psychological need satisfaction in the leisure context, a social environment that is of particular importance during adolescence, is warranted. There is a growing body of literature demonstrating the influence of participation in leisure activities on the positive development and well-being of adolescents. The strongest correlations have been found between participation in organized activities, school well-being, and academic achievements (Eccles et al. 2003; Fredricks and Eccles 2008; Fredricks and Eccles 2006; Gilman 2001). Others have found leisure participation during adolescence to be related to low levels of depressed mood and anxiety (Larson et al. 2002), and of aggression, antisocial behavior, and crime (Rhodes and Spencer 2005). Leisure activities may thus promote positive health as well as prevent problem behavior among adolescents, but more research is needed to explain the operating mechanisms in these relationships.

The developmental opportunities gained through satisfaction of essential psychological needs within leisure activities may represent one explanatory mechanism for the positive effects of participation in activities on well-being. The significance of need satisfaction for the well-being of adolescents has been found for other important domains in their lives, such as the family, the school, and in peer relations (Milyavskaya et al. 2009; Sheldon et al. 2009; Veronneau et al. 2005). However, to the authors’ knowledge, no previous studies have investigated the impact of the psychological needs for competence, relatedness, and autonomy (Ryan and Deci 2000) experienced in the context of adolescents leisure activities and the effect these have on their general life satisfaction. Because participation in leisure time activities constitutes an important part of adolescents’ lives, it is essential to understand better how such participation may contribute to young people’s life satisfaction through leisure specific need satisfaction. The present study therefore sets out to study the underlying association between need satisfaction and the observed positive outcomes of adolescents’ participation in leisure activities. Better understanding of such mechanisms may be used then as a basis for developing the approaches taken in organized leisure time activities to ensure that they contribute to young people’s development and enjoyment in the best possible ways.

### Basic Psychological Needs and Well-being

Perceived life satisfaction, referred to as the global, cognitive judgments of one’s life (Pavot et al. 1991), is considered to be an important indicator of positive psychological well-being (Huebner et al. 2005). According to self-determination theory there are some basic needs that must be satisfied in order to promote psychological well-being and secure healthy development (Deci and Ryan 2000). These are the psychological needs for competence, relatedness, and autonomy, which are postulated to be universal across people and cultures and important in all domains and aspects of a person’s life (Deci and Ryan 1985; Ryan and Deci 2000). Participation in leisure activities among adolescents constitutes one such domain where basic psychological needs may be satisfied.

The need for competence involves feeling effective in interacting with the environment, experiencing opportunities to use one’s capacities, and managing tasks that are challenging (Deci and Ryan 1985; Ryan and Deci 2000; White 1959). During leisure time activities, young people have opportunities to be good at and/or feel good about something, be challenged, and develop their skills. When the need for competence is satisfied, individuals may gain psychological rewards, and feel that they can act effectively and bring about goals. The need for competence is related closely to the need for relatedness, because feedback from significant others may be an important contributor to adolescents’ feelings of competence (Fredricks et al. 2002). Relatedness refers to the feeling of being connected to others, which includes relating to and caring for others, feeling cared for by those others, and feeling involved with the social world more generally (Baumeister and Leary 1995). Because adolescence is a period of transition, with declining conformity to parents, social relationships with peers and other adults may become increasingly important, both for general psychological well-being and for enjoyment of activities. Involvement in leisure activities links an adolescent to a set of similar peers, provides shared experiences and goals, and can reinforce friendships between peers and relationships with adults (Eccles et al. 2003). Consequently, leisure activities may facilitate adolescents’ developmental needs for social relatedness, and can contribute to one’s identity as an important and valued member of a group or community. In such activities adolescents have opportunities to be with other people, cooperate with them, and feel respected and liked.

The need for autonomy refers to people’s strivings to feel that one’s activities are self-chosen and concordant with intrinsic interests, and to have a voice or input in determining one’s own behavior (Deci and Ryan 1985). For adolescents, participation in leisure activities is most likely voluntary, self-initiated and based on their own choices and interests. Although social–cultural values and opportunities may influence participation, leisure is likely to be a context for varying degrees of self-determined and autonomous behavior. In leisure environments that support adolescents’ need for autonomy, young people may have opportunities to be involved in decision making, for example to choose their own musical pieces or types of music, influence the strategy of a team, and/or suggest positions in a sporting game. Activity leaders are important in facilitating opportunities for autonomy satisfaction, in part through the extent to which they involve participants in decisions.

### Participation in Leisure Activities and Developmental Outcomes

Participation in leisure activities may provide adolescents with unique developmental opportunities for socialization and learning. In addition, participation in leisure activities has been linked in several studies with well-being and good mental health (e.g. Casey et al. 2005; Fletcher et al. 2003; Larson 2000), and may be associated more specifically with satisfaction of competence, relatedness, and autonomy. The satisfaction of these three psychological needs in leisure activities potentially may depend on the extent of adolescents’ involvement in the activities. Earlier studies have found that factors such as breadth and intensity of participation in activities were both associated with positive developmental outcomes, such as academic adjustment, psychological competencies, and positive peer context (for an overview see Bohnert et al. 2010). The assumed mechanism is that increased participation provides youth with a wider range of activities related to growth experiences, increased levels of socialization experiences, and larger support networks. Similar mechanisms also have been proposed through activity theory (Brajsa-Zganec et al. 2011; Lemon et al. 1972; Rodriguez et al. 2008). In addition, the link between the amount of participation in leisure activities and the level of positive experiences has been supported in previous studies of adolescents (Hansen and Larson 2007; Marsh and Kleitman 2002).

### Need Satisfaction in Domains of the Lives of Adolescents

According to Eccles et al. (1993), and to the stage-environment fit perspective, there is a change in developmental needs during adolescence and a mismatch may arise between the needs of developing adolescents and the opportunities afforded by their social environments. In the self-determination theory (SDT) tradition, the focus of research has not been centered on adolescents in particular. Therefore, further research on their experiences of psychological need satisfaction within important domains of adolescent life is warranted. Several previous studies have addressed psychological need satisfaction in children and adolescents by focusing on specific settings, such as the school and the family environment. Veronneau et al. (2005) examined the unique contribution of the three psychological needs and concurrent and future levels of well-being in a sample of 331 students in the third and seventh grades. These authors found that, in the domains of school and family, all three needs were associated with well-being and that the psychological need for competence seemed to be particularly important. No positive associations were found between need satisfaction with friends and well-being, which may be due to the somewhat younger ages of the participants, as peers become increasingly important during adolescence. In a recent study by Milyavskaya et al. (2009), the authors investigated an older group (mean age, 14.5 years) of 720 adolescents from the US, Canada, and France. They found that need satisfaction experienced with friends, at home, at school, and in part-time jobs was positively related to well-being, which was evident across the three countries. These findings also were replicated in a sample of Chinese adolescents within the same study. Furthermore, Sheldon et al. (2009) also tested the universality of psychological needs regarding autonomy, competence, and relatedness in the school setting in samples of 363 Nigerian students (mean age, 14.2 years) and 926 Indian students (mean age, 14.4 years). The authors found that satisfaction of all three needs in the classroom was equally important to general life satisfaction. These previous studies in the home, school and peer setting indicate the importance of testing whether the same mechanisms of psychological need satisfaction for competence, relatedness, and autonomy may explain the observed positive relationship between adolescents’ participation in leisure activities and their life satisfaction.

## The Present Study

The present study sought to extend previous research demonstrating the positive relationship between need satisfaction in different domains and the well-being of children and adolescents (Milyavskaya et al. 2009; Veronneau et al. 2005; Sheldon et al. 2009). Because the leisure activity context is one in which adolescents spend a significant amount of their free time, it was reasoned that perceived satisfaction of the three needs in leisure activities would be associated with general life satisfaction beyond the activity setting, and that each of the three needs experienced in the leisure activity setting would independently relate to increased life satisfaction.

Taken together, theoretical assumptions and previous research justify a path analysis to identify the relationships between participation in leisure activities, basic psychological need satisfaction, and life satisfaction. The model was thus based on the theoretical and empirical assumption that increased participation in activities is associated with greater developmental opportunities (e.g. Bohnert et al. 2010; Hansen and Larson 2007) and increased well-being. Therefore, in the present study it was hypothesized that increased participation in activities during adolescence is associated with higher life satisfaction and that this relationship is explained by the mediation of the positive growth experiences of competence, relatedness, and autonomy within those activities.

Demographic variables, such as socioeconomic status and the sex of the adolescents, are both factors that may influence the abovementioned relationships. Previous research has shown that the socioeconomic status of the family influences participation in activities (e.g. Casey et al. 2005; Villarruel et al. 2005) and life satisfaction (e.g. Ash and Huebner 2001; Burton and Phipps 2008; Levin et al. 2011). Socioeconomic status was therefore controlled for in the model, to examine the relationships over and above this indicator. Furthermore, there is little research that has examined how the relationship between participation in activities and beneficial outcomes may differ between boys and girls (Mahoney et al. 2005). The proposed theoretical model in the present study was thus also tested for differences across sexes.

## Methods

### Sample

Data used in this study were collected in December 2005 through Norwegian participation in the Health Behaviour in School-aged Children (HBSC) study, a World Health Organization (WHO) collaborative cross-national survey. For the analysis, we used a representative sample of 3,273 Norwegian students aged 15 (*N* = 1,537) and 16 years (*N* = 1,736), with approximately equal numbers of boys and girls (51.8 % boys). Participants were selected using a stratified systematic sampling procedure, thus providing a nationally representative sample (Roberts et al. 2009). The sampling unit was the school class (15-year-old children attended the last year of compulsory lower secondary school, and 16-year-old children attended the first year of upper secondary school), with one participating class per age group. Of the 286 invited schools, 70 % chose to participate in the study and of these the student response rate was 83 %. When taking into account the nonparticipating schools, there was a response rate of 58 % at the student level.

### Procedure

Data were collected in accordance with a standardized protocol in the HBSC (Roberts et al. 2009). Prior to the survey, school principals gave informed consent, while passive consent was obtained from parents, who were able to withdraw their child(ren) from the study. Students were informed that participation was voluntary and their responses would be anonymous. Instructions were given to teachers on how to administer the survey. Questionnaires were given to the participating students in the selected classes and were filled out during an ordinary school period (45 min). Each student put the completed questionnaire in a sealed envelope before handing it in. All data were treated anonymously. Data collection procedures were supervised by Regional Committees for Medical Research Ethics and approved by the Norwegian Social Science Data Services Privacy Ombudsman for Research.

### Measures

#### Life Satisfaction

Life satisfaction was measured using Huebner’s Student Life Satisfaction Scale (SLSS) (Huebner 1991a). This is a global self-report measure developed for children in Grades 3–12. The SLSS measures students’ internal, reflective appraisals of reality. It takes a broad view of the life satisfaction concept and requires respondents to make overall life assessments that are not related to specific domains (Huebner et al. 2004). The present study used the nine-item version of the scale, which contains nine statements about well-being with a four-point agreement scale (“never”, “sometimes”, “often” or “almost always”). Examples of statements are “I like the way things are going for me”, “My life is going well”, and “I would like to change many things in my life”. The scale has shown good psychometric properties and support for construct validity in a range of studies (e.g. Huebner 1991a, b; Terry and Huebner 1995). The scale had a Cronbach’s alpha of .90 in the current study, indicating high internal consistency.

#### Basic Need Satisfaction in Leisure Activities

To assess perceived need satisfaction in participation in leisure activities, a subset was adapted from the Basic Need Satisfaction at Work Scale (Deci et al. 2001; Ilardi et al. 1993), as at the time of protocol development and data collection no other relevant scale applying specifically to the leisure activity context of adolescents was available. Three subscales were used: competence, relatedness, and autonomy. There were three items for competence (e.g. “I learn interesting new things in the activities I do in my leisure time”), three items for relatedness (e.g. “The people I spend time with in my leisure time activities I consider to be my friends”), and three items for autonomy (e.g. “I feel free to express my ideas and opinions in my leisure time activities”). Respondents were asked to think about the leisure activities that they liked to do best when they responded to the items. The adolescents responded on a five-point Likert scale from “strongly agree” to “strongly disagree”. Cronbach’s alpha for the competence, relatedness, and autonomy subscales was .70, .79, and .52, respectively.

#### Participation in leisure activities

Participation in leisure activities was measured using a list of 22 leisure activities (e.g. soccer, handball, karate, swimming, cross-country skiing, athletics, gymnastics, choir, brass band, playing an instrument, drama, dancing, and scouting). Adolescents reported how often they participated in each of the activities, either through organizations or self-initiated participation: “don’t do this activity” (0), “two to three times a month or more seldom” (1), “about once a week” (2), or “two times a week or more” (3). A leisure activity variable was constructed based on the sum score of all 22 activities and represented a measure of general participation in activities. Thus, this composite variable represented both the level of general participation and increased involvement in additional activities.

#### Socioeconomic Status

The Family Affluence Scale (FAS), which is a measurement of access to material goods in the family (Currie et al. 2008a) was used to assess socioeconomic status. FAS is calculated through four questions: “Does your family have a car or a van?” (“no”, “yes, one”, “yes, two or more”), “How many computers does your family own?” (“none”, “one”, “two”, “more than two”), “Do you have your own bedroom?” (“no”, “yes”), and “During the past 12 months, how many times did you travel away on holiday with your family?” (“not at all”, “once”, “twice”, “more than twice”) (Currie et al. 2008a). The four indicators were combined to produce a linear composite score of family affluence ranging from zero (lowest affluence) to nine (highest affluence). Several studies have shown the FAS to be a valid and valuable indicator of family wealth, easily reported by youth, and it has been strongly associated with a number of individual-level health behaviors and outcomes (Andersen et al. 2008; Currie et al. 1997, 2008b; Holstein et al. 2004; von Rueden et al. 2006).

### Analyses

For the initial descriptive analyses, and Pearson correlations, IBM SPSS version 19.0 (SPSS Inc., Armonk, NY) was used. Next, confirmatory factor analysis using AMOS program 18.0 (Arbuckle 2009) was performed to examine the factor structure of the three-factor measure of perceived need satisfaction for competence, relatedness, and autonomy. Structural equation modeling (SEM) in AMOS was further used to examine the interrelationships between the study variables and to test the mediation hypothesis. The SEM analysis was predominantly confirmatory, because the theoretical rationale was regarded quite important, and the goal was to provide a quantitative evaluation of the hypothesized theoretical model (cf. Kline 2005). Life satisfaction and satisfaction with competence, relatedness, and autonomy were treated as latent continuous variables, while participation in activities and family affluence were observed variables in the model. To test the hypothesized model, the measurement model and the structural model were tested. The covariance matrix of the hypothesized model was analyzed using maximum likelihood estimation. Because Chi-square is highly sensitive to sample size, the global model fit was estimated by a comparative fit index (CFI) and the root mean square error of approximation (RMSEA) (Browne and Cudeck 1993; Byrne 2001). Recommended cut-off criteria for the two fit indices are CFI greater than .95, and RMSEA equal to or below .05 (indicating good model fit) (Hu and Bentler 1999). The structural model also was tested through a multigroup analysis (Byrne 2001, 2004), to test whether the model varied between sexes. Sobel test was used to assess whether the study variables of competence, relatedness, and autonomy satisfaction were significant mediators in the association between participation in activities and life satisfaction (Preacher and Leonardelli 2011; Sobel 1982).

## Results

### Descriptive Statistics and Correlations

Table 1 show that there was no non-normality in the data. Univariate skewness of 2.0 and higher and kurtosis of 7.0 and higher is considered moderate to high nonnormality and has been found to create problems in analyses (Curran et al. 1996; Muthen and Kaplan 1985, 1992). All variables in the present study had values well below these levels. When comparing the levels of the three needs, the reported mean level of autonomy satisfaction was significantly higher than the reported competence satisfaction (Cohen’s *d* = .54) and relatedness satisfaction (Cohen’s *d* = .19).

**Table 1:** Descriptive statistics including minimum and maximum scores, mean, standard deviation (SD), skewness/SE, and kurtosis/SE (*N* = 3,270)

	Min.–max.	Mean	SD	Skew	SE	Kurt.	SE
1. Family affluence	0–9	6.69	1.65	−.53	.04	−.08	.09
2. Activity participation	0–40	7.62	5.37	1.04	.05	1.82	.10
3. Competence	3–15	11.91	2.18	−.75	.04	1.17	.09
4. Relatedness	3–15	12.61	2.17	−1.05	.04	1.43	.09
5. Autonomy	3–15	12.99	1.78	−1.17	.04	.93	.09
6. Life satisfaction	9–36	26.51	5.78	−.57	.04	−.35	.09

To determine whether there were significant sex differences in the means of the study variables, we conducted independent samples *t* tests for equality of means. Boys reported higher levels of life satisfaction (Cohen’s *d* = .41), competence satisfaction (Cohen’s *d* = .13), and participation in leisure activities (Cohen’s *d* = .18) (Table 2). There were no statistically significant sex differences in relatedness and autonomy satisfaction.

**Table 2:** Descriptive statistics for study variables, by sex, and t tests for sex differences

	Boys			Girls			*t*	*df*	*p*
	*M*	SD	*N*	*M*	SD	*N*			
Activity participation	8.09	5.61	1,129	7.15	5.09	1,153	4.19	2,280	.000
Competence	12.05	2.22	1,581	11.77	2.14	1,478	3.55	3,057	.000
Relatedness	12.58	2.16	1,564	12.63	2.18	1,471	−71	3,033	.481
Autonomy	13.04	1.80	1,563	12.94	1.77	1,473	1.51	3,034	.131
Life satisfaction	27.62	5.36	1,573	25.33	5.97	1,464	11.11	2,942	.000

Correlation analyses of all study variables, presented in Table 3, show that the relationships between life satisfaction and the predictor variables were all positive and significant at the *p* < .001 level. Of the three psychological needs, satisfaction of competence need in leisure activities had the strongest correlation with life satisfaction (*r* = .33).

**Table 3:** Bivariate correlations among subscales and dependent and independent variables

	1	2	3	4	5	6
1. Family affluence	–					
2. Activity participation	.14***	–				
3. Competence	.12***	.26***	–			
4. Relatedness	.13***	.20***	.55***	–		
5. Autonomy	.09***	.10***	.50***	.49***	–	
6. Life satisfaction	.18***	.15***	.33***	.29***	.23***	–

*** *p* < .001

### SEM Analysis

#### Measurement Model

Confirmatory factor analysis (CFA) indicated the following fit for the three-factor measure of perceived need satisfaction (consisting of the three latent factors competence, relatedness, and autonomy): CFI = .946 and RMSEA = .075 (90 % CI = .069–.081). According to Byrne (2001), these may be regarded as acceptable fit indices. Table 4 presents the factorial structures of the study variables competence, relatedness, and autonomy, and, as shown here, all factor loadings were above .30, which is commonly used to define a “salient” factor loading (Brown 2006).

**Table 4:** Standardized factor loadings (SFA) for the three basic needs

Variables	Items	SFA
Competence satisfaction	COMP1	.67
	COMP2	.64
	COMP3	.68
Relatedness satisfaction	REL1	.74
	REL2	.74
	REL3	.76
Autonomy satisfaction	AUT1	.37
	AUT2	.58
	AUT3	.65

All variables in the measurement model were allowed to covary. The error terms of two of the single observed indicators in the life satisfaction variable were also allowed to covary, because these two items were the only ones addressing negative perceptions of life. This procedure was also performed in a study by Danielsen et al. (2009) using the same latent life satisfaction scale (Huebner 1991a) as in the present study. The existence of such a negativity factor has been well established in the research literature (DiStefano and Motl 2006; Marsh 1996; Springer and Hauser 2006). The results indicated the following fit indexes: CFI = .960; RMSEA = .045 (90 % CI = .043–.047). According to cut-off criteria recommended by Hu and Bentler (1999), this may be regarded as a good fit of the model.

#### Structural Model

**Fig. 1:**
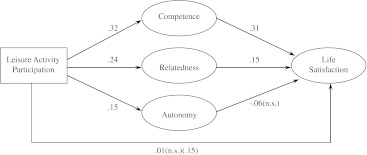
Structural model of the relation between participation in leisure activities, the three needs for autonomy, competence, relatedness, and life satisfaction, with standardized regression and correlations, all paths *p* < .001 unless indicated not significant (n.s.), controlled for family affluence. Direct effect of leisure activity participation on life satisfaction in parenthesis

The proposed path model was tested through structural equation modeling (SEM) analysis, assessing direct effects between (1) participation in activities and life satisfaction, (2) participation in activities and the three basic needs, and (3) basic needs and life satisfaction; and assessing (4) the indirect effect of participation in activities on life satisfaction mediated by the basic needs in leisure activities. The error terms of the three latent factors competence, relatedness, and autonomy were allowed to covary. This makes sense conceptually and theoretically, because the levels of need satisfaction across the three needs tend to be highly correlated in a particular situation (Veronneau et al. 2005), and because need satisfaction is largely contextually determined (Deci and Ryan 2008). The goodness-of-fit indices for the structural model were as follows: CFI = .957, RMSEA = .045 (90 % CI = .043–.048). Figure 1 displays standardized regression weights and correlations for this structural model. Most of the results were significant at the *p* < .01 level. Eighteen percent of the variance in life satisfaction was accounted for by the predictor variables. As indicated in the figure, the strongest parameters in the structural model were the direct relationship between participation in leisure activities and competence satisfaction (.32) and the direct relationship between competence satisfaction and life satisfaction (.31). Participation in leisure activities was directly and strongly associated with relatedness satisfaction (.24) and modestly associated with autonomy satisfaction (.15). Furthermore, relatedness satisfaction was modestly related to life satisfaction, whereas there was no significant association between autonomy satisfaction and life satisfaction. The SEM model did not reveal a significant direct association between participation in leisure activities and life satisfaction. The structural model was also tested using a multigroup analysis approach to test whether the model was invariant between sexes (Byrne 2004). The path coefficients were shown to be invariant across the two sex groups (with CFI changing ≥.002) (Meade et al. 2008), meaning there were no significant differences between boys and girls in any of the respective paths tested.

The Sobel test showed that both competence satisfaction (*z* = 4.07, *p* < .001) and relatedness satisfaction (*z* = 2.27, *p* < .05) fully mediated the participation in activities–life satisfaction relationship. Autonomy satisfaction had no such mediation effect.

## Discussion

The current study extends previous support for the well-being benefit of need satisfaction in core developmental settings for adolescents—school, family, and peers (Milyavskaya et al. 2009; Veronneau et al. 2005; Sheldon et al. 2009)—to the context of leisure activities. In line with the self-determination theory (SDT) (Deci and Ryan 2000) the findings demonstrate that the relationship between participation in leisure activities and life satisfaction is mediated by the psychological need satisfaction within participation among adolescents, a rather under-investigated group in the SDT tradition. In a sample of more than 3,000 adolescents in Norway satisfaction of competence and relatedness activities fully mediated the relationship between general participation in activities and life satisfaction. This indicates that these two needs are of particular importance for adolescents within the leisure activity context. Moreover, the extent to which level of participation relates to adolescents’ life satisfaction may be explained through the positive processes of increased need satisfaction for competence and relatedness following participation. The operant mechanisms may be that the more adolescents participate in leisure activities, the more opportunities for basic need satisfaction they get, and this may have a higher positive effect on their life satisfaction. In general, the results of the present study were consistent with the assumptions of self-determination theory and add to previous research investigating other important contexts for adolescents’ need satisfaction. The findings thus suggest that need satisfaction in the context of leisure activities may be an important source of well-being for adolescents. The fact that the model was invariant across sexes strengthens these assumptions.

Leisure activities are most often peer based and usually involve social relations with peers. From the literature it is known that peer relations become increasingly important especially during the years of adolescence. The finding by Milyavskaya et al. (2009) about the positive relationship between need satisfaction with friends and well-being, as well as the results of the current study demonstrate that need satisfaction with peers and friends plays an important role in well-being at this stage of life, and that need satisfaction occurs in leisure activity involvement with peers, as well as in the school and the family (Milyavskaya and Koestner 2011; Sheldon et al. 2009; Veronneau et al. 2005).

All three needs made independent significant contributions to adolescents’ life satisfaction. This is consistent with the assumptions proposed in self-determination theory about all three needs being important for individuals’ well-being (Deci and Ryan 2000). However, when comparing the correlations and contributions of the three needs to life satisfaction in the leisure activity setting, competence and relatedness satisfaction seemed to be the most important psychological needs for adolescents’ life satisfaction. The structural model further showed that, when controlling for the other needs, the path between autonomy satisfaction and life satisfaction did not remain significant. This means that only competence and relatedness satisfaction in leisure activities fully explained the association between level of participation in activities and life satisfaction. Competence satisfaction was the strongest mediator in the present model. These findings suggest that experiencing satisfaction for competence (in particular) and relatedness in the leisure activity setting is of higher importance for adolescents’ life satisfaction than feeling autonomous in this setting. Similar findings have been found in other important domains in the lives of children and adolescents. On the basis of the results in the study by Veronneau et al. (2005) they suggested that competence is the most important psychological need in middle childhood and early adolescence. However, it is well established in the literature that there is an increased need to feel autonomous in adolescence and this may be especially salient at a time of many transitions (Eccles et al. 1993). The lack of mediation found for autonomy satisfaction in the current study may imply simply that increased participation in activities does not necessarily increase the feeling of autonomy to the same degree as it does for competence and relatedness, and consequently it has less effect on life satisfaction. Experiences of competence and relatedness satisfaction, on the other hand, possibly may have higher potentials to be enhanced with increased levels of participation. Feeling autonomous may be also something that is implicit in the adolescents own choices of activities, and as demonstrated by the initial analyses in the present study, autonomy satisfaction in the leisure activity setting is still of statistical significance for the adolescents life satisfaction.

### Participation in Leisure Activities by Adolescents

The findings of the present study showed that the experiences of need satisfaction of boys and girls were generally high for all three needs. This is a positive result, but this result may also be due to the targeted age of the participants. From early years, children undertake different kinds of leisure activities; however, by the age of 15 and 16, most individuals have probably finished testing new activities and may seek, to a stronger degree, participation in activities that they know will satisfy their needs, thereby choosing activities according to interest and talent.

The present study investigated the positive impact of participation across a range of activities. The variable was constructed in such a way that increased levels of participation also meant participation in more activities. Although not strictly measured as intensity or breadth of activities, according to Bohnert et al. (2010) the measure used in the current study still may be considered a combination of both. The results then imply that the more often adolescents participate, and the more activities they participate in, the higher their experience of need satisfaction and the higher their life satisfaction. In the current model, participation in activities and need satisfaction explained 18 % of the variance for life satisfaction. Such a finding makes conceptual sense because the leisure context represents only one of the important domains in the lives and well-being of adolescents. Additionally, life satisfaction was assessed at the global level whereas basic need satisfaction and participation in activities were measured at the contextual level (see, for example, Vallerand 1997; Vallerand and Ratelle 2002).

High levels of participation in leisure activities may not have exclusively positive effects on individual well-being, but also may have negative effects. In a sample of adults, Stenseng et al. (2011) found that obsessive passion for a leisure activity was correlated negatively with basic need satisfaction and correlated positively with negative affective outcomes of activity engagement. The overscheduling hypothesis assumes that children and adolescents are overburdened with activities and this supposedly threatens their well-being (Mahoney et al. 2008). However, studies mostly have failed to support this theory (see Bohnert et al. 2010). Based on studies investigating participation by children and young people in various organized activities, Mahoney et al. (2008) concluded that approximately 60 % participate in such activities, and that fewer than one in 10 could be considered overscheduled. This suggests that the threshold level is very high, and that increased levels of participation generally and usually have a positive, linear effect on well-being. The focus of attention, therefore, should be on promoting participation among those who do not participate, rather than on the very few who are overscheduled.

### Limitations

The present study is based on cross-sectional data, which measure all correlations at one point in time only and do not permit claims of causality. Based on the model tested in this study and findings from previous empirical work, it is likely that participation in activities and need satisfaction affect life satisfaction, rather than the reverse. Longitudinal studies allow predictions to be made about the causes and effects of participation in leisure activities and possible positive effects. Such research designs also may provide opportunities to control for factors that may confound relationships. However, the demonstration of significant relationships between participation, need satisfaction, and life satisfaction across sexes, when controlling for socioeconomic status, may strengthen the assumptions that there are indeed elements in participation, possibly beyond such socioeconomic and individual factors, that may increase adolescents’ life satisfaction. The rather large sample size may have influenced model fit indexes and the significance of weak relationships. Therefore, caution is needed in the interpretation of the results.

The current study was based on self-reports and so the experiences of autonomy satisfaction, for instance, do not necessarily reflect actual satisfaction of autonomy in the leisure setting. Results of the present study, therefore, should be treated with caution. In previous studies, the measurement of autonomy also has been reported as difficult, especially tapping into the “true” content of autonomy in line with how it is defined in self-determination theory. The reliability of the autonomy subscale was relatively low in the present study. Thus, the autonomy measure used in the present study may not have been ideal for the leisure setting and the adolescence age group. However, low alpha values for autonomy also have been found in previous studies (Deci et al. 2001). Additionally, the confirmatory factor analysis performed in the present study indicated an acceptable fit for the three-factor measure of perceived need satisfaction (competence, relatedness, and autonomy), with adequate factor loadings (Brown 2006). We may therefore assume that the need satisfaction measures used in the analysis most likely represent the adolescents’ experiences of satisfaction for autonomy, competence and relatedness within leisure activities. Previous research has shown that the experiences within participation in activities may differ between activities (Hansen et al. 2010; Larson et al. 2006; Tinsley and Eldredge 1995), and some activities even may thwart needs instead of satisfying them (Vallerand and Ratelle 2002). It is, therefore, likely that not all activities in the present study provided equal opportunities for individuals’ need satisfaction. The aggregated measure of participation in leisure activities used in the study comprises many different activities. Participation in several activities potentially may compensate for reduced satisfaction of some needs in certain activities. The results give a broad picture of how participation in activities, need satisfaction within such activities, and adolescents’ life satisfaction are related, which was the main aim of the current study. The assumptions about these mechanisms also are strengthened by the fact that the study employs a nationally representative sample. Thus, one may assume that the findings provide a plausible picture of adolescents’ participation in leisure time activities in Norway and of their positive leisure experiences and levels of well-being.

## Conclusions and Implications

The results of the present study showed that psychological need satisfaction experienced in the leisure activity domain is associated positively with adolescents’ increased life satisfaction. Furthermore, need satisfaction fully mediated the association between participation in activities and life satisfaction, implying that it is the positive processes of need satisfaction, and especially the satisfaction of competence and relatedness that seem to benefit adolescent boys’ and girls’ general life satisfaction. These findings add to previous research investigating the positive impact of need satisfaction in other important domains in the lives of children and adolescents.

The current findings also may add new and important knowledge for the planning and organization of activities, focusing on promoting a positive climate within activities that supports the essential psychological needs of children and adolescents. Leisure activities that provide adolescents with opportunities for skill development, allow them to feel that they are good at something, give them an active contributory role, and focus on social relations and positive interactions between participants, may promote growth, development, and an increased subjective well-being in the lives of adolescents. Further, the experience of need satisfaction within leisure activities may be important for individuals to continue the activity, and programs that fail to satisfy the three needs may lead to withdrawal from both current and future participation in leisure activities (e.g. Calvo et al. 2010; Eccles 2005; Sarrazin et al. 2002). Because need satisfaction within leisure activities is demonstrated to have beneficial effects on adolescents’ well-being, it is also important to promote sustained participation to benefit their positive development and their mental (and physical) health.

Because previous research has found that variables such as self-efficacy influence basic-need satisfaction (Moen and Skaalvik 2008; Moen et al. 2009) and life satisfaction (Danielsen et al. 2009; Leganger et al. 2000), it would be valuable to take into account such potential confounding variables as positive self-perceptions in future research. Future research also needs to target specific activities and to map the experience of need satisfaction within different types of activities in order to gain knowledge about how various activities provide a supportive environment where the needs for competence, relatedness, and autonomy may be met. This may provide practitioners and stakeholders of activities with important and more detailed information to guide appropriate action regarding planning and organization of activities for children and adolescents to stimulate participation and prevent drop out.

## References

[CR1] Andersen A, Krolner R, Currie C, Dallago L, Due P, Richter M (2008). High agreement on family affluence between children’s and parents’ reports: International study of 11-year-old children. Journal of Epidemiology and Community Health.

[CR2] Arbuckle JL (2009). Amos 18.0 user’s guide.

[CR3] Ash C, Huebner ES (2001). Environmental events and life satisfaction reports of adolescents: A test of cognitive mediation. School Psychology International.

[CR4] Baumeister RF, Leary MR (1995). The need to belong: Desire for interpersonal attachments as a fundamental human motivation. Psychological Bulletin.

[CR5] Bohnert A, Fredricks J, Randall E (2010). Capturing unique dimensions of youth organized activity involvement: Theoretical and methodological considerations. Review of Educational Research.

[CR6] Brajsa-Zganec A, Merkas M, Sverko I (2011). Quality of life and leisure activities: How do leisure activities contribute to subjective well-being?. Social Indicators Research.

[CR7] Brown TA (2006). Confirmatory factor analysis for applied research.

[CR8] Browne MW, Cudeck R, Bollen KA, Long JS (1993). Alternative ways of assessing model fit. Testing structural equation models.

[CR9] Burton P, Phipps S (2008). Economic resources, relative socioeconomic position and social relationships: Correlates of the happiness of young Canadian teens. Child Indicators Research.

[CR10] Byrne B (2001). Structural equation modeling with AMOS: Basic concepts, applications, and programming.

[CR11] Byrne B (2004). Testing for multigroup invariance using AMOS graphics: A road less traveled. Structural Equation Modeling.

[CR12] Calvo TG, Cervello E, Jimenez R, Iglesias D, Murcia JAM (2010). Using self-determination theory to explain sport persistence and dropout in adolescent athletes. Spanish Journal of Psychology.

[CR13] Casey DM, Ripke MN, Huston AC (2005). Activity participation and the well-being of children and adolescents in the context of welfare reform.

[CR14] Curran PJ, West SG, Finch JF (1996). The robustness of test statistics to nonnormality and specification error in confirmatory factor analysis. Psychological Methods.

[CR15] Currie CE, Elton RA, Todd J, Platt S (1997). Indicators of socioeconomic status for adolescents: The WHO health behaviour in school-aged children survey. Health Education Research.

[CR16] Currie C, Molcho M, Boyce W, Holstein B, Torsheim T, Richer M (2008). Researching health inequalities in adolescents: The development of the health behaviour in school-aged children (HBSC) family affluence scale. Social Science and Medicine.

[CR17] Currie, C., Nic Gabhainn, S., Godeau, E., Roberts, C., Smith, R., Currie, D., et al. (Eds.). (2008). *Inequalities in young people’s health*. HBSC International Report from the 2005/2006 Survey. Health Policy for Children and Adolescents, No. 5. Copenhagen, Denmark: WHO Regional Office for Europe.

[CR18] Danielsen AG, Samdal O, Hetland J, Wold B (2009). School-related social support and students’ perceived life satisfaction. The Journal of Educational Research.

[CR19] Deci EL, Ryan RM (1985). Intrinsic motivation and self-determination in human behavior.

[CR20] Deci EL, Ryan RM (2000). The “what” and “why” of goal pursuits: Human needs and the self-determination of behavior. Psychological Inquiry.

[CR21] Deci EL, Ryan RM (2008). Facilitating optimal motivation and psychological well-being across life’s domains. Canadian Psychology-Psychologie Canadienne.

[CR22] Deci EL, Ryan RM, Gagne M, Leone DR, Usunov J, Kornazheva BP (2001). Need satisfaction, motivation, and well-being in the work organizations of a former Eastern bloc country: A cross-cultural study of self-determination. Personality and Social Psychology Bulletin.

[CR23] DiStefano C, Motl RW (2006). Further investigating method effects associated with negatively worded items on self-report surveys. Structural Equation Modeling.

[CR24] Eccles JS (2005). Studying the development of learning and task motivation. Learning and Instruction.

[CR25] Eccles JS, Roeser RW, Lerner RM, Steinberg L (2009). Schools, academic motivation, and stage-environment fit. Handbook of adolescent psychology.

[CR26] Eccles JS, Roeser RW (2011). Schools as developmental contexts during adolescence. Journal of Research on Adolescence.

[CR27] Eccles JS, Midgley C, Wigfield A, Buchanan CM, Reuman D, Flanagan C (1993). Development during adolescence: The impact of stage-environment fit on young adolescents experiences in school and in families. American Psychologist.

[CR28] Eccles JS, Barber BL, Stone M, Hunt J (2003). Extracurricular activities and adolescent development. Journal of Social Issues.

[CR100] Fletcher, A. C., Nickerson, P., & Wright, K. L. (2003). Structured leisure activities in middle childhood: Links to well-being. *Journal of Community Psychology,**31*(6), 641–659.

[CR29] Fredricks JA, Eccles JS (2006). Is extracurricular participation associated with beneficial outcomes? Concurrent and longitudinal relations. Developmental Psychology.

[CR30] Fredricks JA, Eccles JS (2008). Participation in extracurricular activities in the middle school years: Are there developmental benefits for African American and European American youth?. Journal of Youth and Adolescence.

[CR31] Fredricks JA, Alfeld-Liro CJ, Hruda LZ, Eccles JS, Patrick H, Ryan AM (2002). A qualitative exploration of adolescents’ commitment to athletics and the arts. Journal of Adolescent Research.

[CR32] Gilman R (2001). The relationship between life satisfaction, social interest, and frequency of extracurricular activities among adolescent students. Journal of Youth and Adolescence.

[CR33] Hansen DM, Larson RW (2007). Amplifiers of developmental and negative experiences in organized activities: Dosage, motivation, lead roles, and adult-youth ratios. Journal of Applied Developmental Psychology.

[CR34] Hansen DM, Skorupski WP, Arrington TL (2010). Differences in developmental experiences for commonly used categories of organized youth activities. Journal of Applied Developmental Psychology.

[CR35] Holstein, B., Parry-Langdon, N., Zambon, A., Currie, C., & Roberts, C. (2004). Socio-economic inequalities and health. In C. Currie, C. Roberts, A. Morgan, R. Smith, W. Settertobulte, O. Samdal, & V. B. Rasmussen (Eds.), *Young people’s health in context. Health behaviour in school*-*aged children (HBSC) study*. International Report from the 2001/2002 Survey. Health policy for children and adolescents, No. 4 (pp. 165–172). Copenhagen, Denmark: WHO Regional Office for Europe.

[CR36] Hu LT, Bentler PM (1999). Cutoff criteria for fit indexes in covariance structure analysis: Conventional criteria versus new alternatives. Structural Equation Modeling.

[CR37] Huebner ES (1991). Initial development of the students’ life satisfaction scale. School Psychology International.

[CR38] Huebner ES (1991). Further validation of the students’ life satisfaction scale: The independence of satisfaction and affect ratings. Journal of Psychoeducational Assessment.

[CR39] Huebner ES, Suldo SM, Smith LC, McKnight CG (2004). Life satisfaction in children and youth: Empirical foundations and implications for school psychologists. Psychology in the Schools.

[CR40] Huebner ES, Valois RF, Paxton R, Drane WJ (2005). Middle school students’ perceptions of their quality of life. Journal of Happiness Studies.

[CR41] Ilardi BC, Leone D, Kasser T, Ryan RM (1993). Employee and supervisor ratings of motivation: Main effects and discrepancies associated with job satisfaction and adjustment in a factory setting. Journal of Applied Social Psychology.

[CR200] Kline, R. B. (2005). *Principles and practice of structural equation modeling*. New York: The Guilford Press.

[CR300] Larson. (2000). Toward a psychology of positive youth development. *American Psychologist,**55*(1), 170–183. 10.1037//0003-066x.55.1.170 11392861

[CR42] Larson RW, Wilson S, Bradford Brown B, Furstenberg FF, Verma S (2002). Changes in adolescents’ interpersonal experiences: Are they being prepared for adult relationships in the twenty-first century?. Journal of Research on Adolescence.

[CR43] Larson RW, Hansen DM, Moneta G (2006). Differing profiles of developmental experiences across types of organized youth activities. Developmental Psychology.

[CR44] Leganger A, Kraft P, Roysamb E (2000). Perceived self-efficacy in health behaviour research: Conceptualisation, measurement and correlates. Psychology and Health.

[CR45] Lemon BW, Bengtson VL, Peterson JA (1972). An exploration of the activity theory of aging: Activity types and life satisfaction among in-movers to a retirement community. Journal of Gerontology.

[CR46] Levin K, Torsheim T, Vollebergh W, Richter M, Davies C, Schnohr C (2011). National income and income inequality, family affluence and life satisfaction among adolescents in 35 countries. Journal of Epidemiology and Community Health.

[CR47] Mahoney JL, Larson RW, Eccles JS, Lord H, Mahoney JL, Larson RW, Eccles JS (2005). Organized activities as development contexts for children and adolescents. Organized activities as contexts of development: Extracurricular activities, after-school and community programs.

[CR48] Mahoney JL, Harris AL, Eccles JS (2008). The over-scheduling myth. Research-to-results brief.

[CR49] Marsh HW (1996). Positive and negative global self-esteem: A substantively meaningful distinction or artifactors?. Journal of Personality and Social Psychology.

[CR50] Marsh HW, Kleitman S (2002). Extracurricular school activities: The good, the bad, and the nonlinear. Harvard Educational Review.

[CR51] Meade AW, Johnson EC, Braddy PW (2008). Power and sensitivity of alternative fit indices in tests of measurement invariance. Journal of Applied Psychology.

[CR52] Milyavskaya M, Koestner R (2011). Psychological needs, motivation, and well-being: A test of self-determination theory across multiple domains. Personality and Individual Differences.

[CR53] Milyavskaya M, Gingras I, Mageau GA, Koestner R, Gagnon H, Fang JQ (2009). Balance across contexts: Importance of balanced need satisfaction across various life domains. Personality and Social Psychology Bulletin.

[CR54] Moen F, Skaalvik E (2008). The triggering effect of business coaching on performance psychology. The International Journal of Coaching in Organizations.

[CR55] Moen F, Skaalvik E, Hacker CM (2009). Performance psychology among business executives in an achievement oriented environment. Journal of Excellence.

[CR56] Muthen B, Kaplan D (1985). A comparison of some methodologies for the factor analysis of non-normal Likert variables. British Journal of Mathematical and Statistical Psychology.

[CR57] Muthen B, Kaplan D (1992). A comparison of some methodologies for the factor-analysis of nonnormal Likert variables: A note on the size of the model. British Journal of Mathematical and Statistical Psychology.

[CR58] Oberle E, Schonert-Reichl KA, Zumbo BD (2011). Life satisfaction in early adolescence: Personal, neighborhood, school, family, and peer influences. Journal of Youth and Adolescence.

[CR59] Pavot W, Diener E, Colvin CR, Sandvik E (1991). Further validation of the satisfaction with life scale: Evidence for the cross-method convergence of well-being measures. Journal of Personality Assessment.

[CR60] Preacher, K. J., & Leonardelli, G. J. (2011). *Calculation of the Sobel test: An interactive calculation tool for mediation tests.* Kristopher J. Preacher, The University of Kansas: http://quantpsy.org/sobel/sobel.htm. Accessed 8 September 2011.

[CR61] Rhodes J, Spencer R (2005). Someone to watch over me: Mentoring programs in the after-school lives of children and adolescents.

[CR62] Roberts C, Freeman J, Samdal O, Schnohr CW, de Looze ME, Gabhainn SN (2009). The health behaviour in school-aged children (HBSC) study: Methodological developments and current tensions. International Journal of Public Health.

[CR63] Rodriguez A, Latkova P, Sun YY (2008). The relationship between leisure and life satisfaction: Application of activity and need theory. Social Indicators Research.

[CR64] Ryan RM, Deci EL (2000). Self-determination theory and the facilitation of intrinsic motivation, social development, and well-being. American Psychologist.

[CR65] Sarrazin P, Vallerand R, Guillet E, Pelletier L, Cury F (2002). Motivation and dropout in female handballers: A 21-month prospective study. European Journal of Social Psychology.

[CR66] Sheldon KM, Abad N, Omoile J (2009). Testing self-determination theory via Nigerian and Indian adolescents. International Journal of Behavioral Development.

[CR67] Sobel M, Leinhart S (1982). Asymptotic confidence intervals for indirect effects in structural equations models. Sociological methodology.

[CR68] Springer KW, Hauser RM (2006). An assessment of the construct validity of Ryff’s scales of psychological well-being: Method, mode, and measurement effects. Social Science Research.

[CR69] Stenseng F, Rise J, Kraft P (2011). The dark side of leisure: Obsessive passion and its covariates and outcomes. Leisure Studies.

[CR70] Terry T, Huebner ES (1995). The relationship between self-concept and life satisfaction in children. Social Indicators Research.

[CR71] Tinsley HEA, Eldredge BD (1995). Psychological benefits of leisure participation: A taxonomy of leisure activities based on their need-gratifying properties. Journal of Counseling Psychology.

[CR72] Vallerand RJ, Hagger MS, Chatzisarantis NLD (1997). A hierarchical model of intrinsic and extrinsic motivation for sport and physical activity. *I*ntrinsic motivation and self-determination in exercise and sport.

[CR73] Vallerand RJ, Ratelle CF (2002). Intrinsic and extrinsic motivation: A hierarchical model.

[CR74] Veronneau MH, Koestner RF, Abela JRZ (2005). Intrinsic need satisfaction and well-being in children and adolescents: An application of the self-determination theory. Journal of Social and Clinical Psychology.

[CR75] Villarruel FA, Montero-Sieburth M, Dunbar C, Outley CW (2005). Dorothy, there is no yellow brick road: The paradox community youth development approaches for Latino and African American urban youth.

[CR76] von Rueden U, Gosch A, Rajmil L, Bisegger C, Ravens-Sieberer U (2006). Socioeconomic determinants of health-related quality of life in childhood and adolescence: results from a European study. Journal of Epidemiology and Community Health.

[CR77] White RW (1959). Motivation reconsidered: The concept of competence. Psychological Review.

